# Facile Synthesis of Ultrahigh Molecular Weight Poly(Methyl Methacrylate) by Organic Halides in the Presence of Palladium Nanoparticles

**DOI:** 10.3390/polym12112747

**Published:** 2020-11-20

**Authors:** Ming Yuan, Lili Xu, Xuetao Cui, Jiaxing Lv, Panpan Zhang, Huadong Tang

**Affiliations:** 1 Institute of Industrial Catalysis, College of Chemical Engineering, Zhejiang University of Technology, Hangzhou 310014, China; 13750872796@163.com (M.Y.); xulili19950516@163.com (L.X.); cxt17816038659@163.com (X.C.); 18846056271@163.com (J.L.); 2 State Key Laboratory of Modern Optical Instrumentation, Zhejiang University, Hangzhou 310027, China; zhpanpan226@126.com

**Keywords:** ultrahigh molecular weight, PMMA, Pd nanoparticles, organic halides, mechanism

## Abstract

A facile and versatile approach for the synthesis of ultrahigh molecular weight poly(methyl methacrylate) (PMMA) at mild conditions was developed. Certain organic halides combined with a catalytical amount of palladium nanoparticles (Pd NPs) were found to be very effective in initiating polymerizations of methyl methacrylate (MMA), methyl acrylate, vinyl acetate and other vinyl monomers. An ultrahigh molecular weight PMMA with a number-average molecular weight of 4.65 × 10^6^ Da and a weight-average molecular weight of 8.08 × 10^6^ Da was synthesized at 70 °C using 2-bromoisobutyric acid ethyl ester (EBiB) as an initiator in the presence of catalytical amount (10.1 ppm) of Pd NPs. A kinetic investigation found that the orders of polymerization with respect to EBiB, Pd NP and MMA were 0.23, 0.50, and 0.58, respectively. Proton nuclear magnetic resonance (^1^H NMR) combined with matrix-assisted laser desorption ionization time of flight mass spectroscopy (MALDI-TOF) and gel permeation chromatography (GPC) were used to prove that the macromolecular chain had an end-group of EBiB residue. The electron spin resonance (ESR), transmission electron microscope (TEM), and X-ray photoelectron spectroscopy (XPS) results reveal that the reaction of EBiB with Pd NPs caused a bromo atom (Br) transfer from EBiB to Pd NPs and resulted in the generation of EBiB residue radical to initiate the polymerization of MMA and the formation of Pd^II^Br_2_ on the surface of Pd nanoparticles.

## 1. Introduction

Poly(methyl methacrylate) (PMMA), also known as acrylic glass or plexiglass, is a transparent, strong, and durable thermoplastic resin with wide applications in aircraft windshields, building windows, furniture decorations, bulletproof screens, signs and displays, sanitary wares, medical materials, LCD screens, and many other uses [1,2]. The mechanic properties of PMMA have long been known to be largely dependent on its molecular weight. For example, the maximum tensile strength for stretched PMMA films with broad molecular weight distributions increased with the increase in molecular weight up to 2.5 × 10^6^ Da, after which the strength tended to increase slowly and finally reached an asymptotic limit at higher molecular weights [3,4]. Therefore, it is of great importance to develop a feasible and facile polymerization approach for the synthesis of ultrahigh molecular weight PMMA.

Traditionally, suspension polymerization and emulsion polymerization have been used to prepare PMMA with high molecular weights in the range of 10^5––^10^6^ Da [5,6,7,8,9]. However, removing the emulsifier and suspension agent from PMMA polymer was complex and costly, and the residual emulsifier and suspension agent in final PMMA products often deteriorate its optical and electronic properties. Osada et al. found that low-pressure plasma produced by electric discharge could initiate the polymerization of methyl methacrylate (MMA) and prepared ultrahigh molecular weight PMMA with viscosity-average molecular weight (*M*_v_) higher than 10^7^ Da [10,11]. Nevertheless, the plasma-initiated polymerization still suffered from many disadvantages, such as slow polymerization rate, the requirement of a high-power radiofrequency generator, and scale-up difficulties [12,13,14]. Recently, a couple of ‘living’/controlled radical polymerization techniques, including atom transfer radical polymerization (ATRP) [15,16,17,18,19,20,21,22,23,24], nitroxide-mediated polymerization (NMP) [25,26], reversible addition-fragmentation chain transfer (RAFT) polymerization [27,28,29,30], and degenerative transfer (DT) have been successfully developed to polymerize MMA and other vinyl monomers [31,32]. Ultrahigh molecular weight PMMA with *M*_n_ = 3.60 × 10^6^ Da (*M*_n_ = number-average molecular weight) and *M*_n_ = 1.25 × 10^6^ Da were synthesized at very high pressure (5000 bar) conditions by ATRP and RAFT, respectively [33,34]. Unfortunately, such a high pressure brings about high production risks and extra scale-up difficulties. Recently, high molecular weight PMMA (*M*_n_ = 10^5^–10^6^ Da) with narrow molecular weight distributions was achieved using aluminum porphyrin and bulky organoaluminum as initiators [35]. Ultrahigh molecular weight PMMA with *M*_n_ up to 1.927 × 10^6^ Da was obtained by frustrated Lewis pair-catalyzed living polymerization using organoaluminum as the corresponding Lewis acid [36], but these organoaluminum compounds are usually moisture- and air-sensitive [37].

Raney metals (Ni, Fe, Co) were found to promote the polymerization of MMA in the presence of CCl_4_ via a radical intermediate [38]. Otsu and coworkers reported that certain activated metals (e.g., Raney Co, reduced Ni) combined with organic halides (CCl_4_, CHBr_3_, CHI_3_, etc) could induce radical polymerizations of MMA [39,40,41]. They found the initial radicals generated from the transferring process of one electron from metal to the carbon–halogen bond of organic halides. These polymerizations showed a relatively slow polymerization rate due to the low activity of activated metals. Palladium nanoparticle (Pd NP) is an effective catalyst for a lot of organic reactions [42,43,44,45,46,47]. In this work, we found that the monodispersed Pd NPs were highly active for the polymerization of vinyl monomers such as MMA, methyl acrylate (MA), vinyl acetate (VAc) and butyl acrylate (BA) in the presence of organic halides and developed a facile and versatile approach for the synthesis of ultrahigh molecular weight PMMA. The kinetics of the polymerization had been investigated in this work and the mechanism of the polymerization was also revealed with a transmission electron microscope (TEM) and electron spin resonance (ESR).

## 2. Materials and Methods

### 2.1. Materials

Methyl methacrylate (MMA, 99%), methyl acrylate (MA, 99%), butyl acrylate (BA, 99%), vinyl acetate (VAc, 99%), 2-bromoisobutyric acid ethyl ester (EBiB, 99%), carbon tetrachloride (CCl_4_, 99%), ethyl 2-bromopropionate (EBP, 99%), *N,N,N’,N’’,N’’*-pentamethyldiethylenetriamine (PMDETA, 99%), benzoyl peroxide (BPO, AR), palladium acetate (AR, Pd 46.0–48.0%), toluene (AR), tetrahydrofuran (THF, >99.5%) and basic aluminum oxide (200–300 mesh) were purchased from Shanghai Aladdin biochemical technology Co., LTD (China). Ethyl α-bromophenylacetate (BPA, 99%), trans indole-3-acrylic acid (98+%) and 1,1-diphenyl-2-trinitrophenylhydrazine (DPPH, 95%) were purchased from Alfa Aesar (Ward Hill, MA, USA). *N*-tert-butyl-alpha-phenylnitrone (PBN, >98%), sodium trifluoroacetate (>98%) and n-dodecyl sulfide were purchased from TCI (Tokyo, Japan). Chloroform-d (99.8%) was purchased from Cambridge Isotope Laboratories, Inc (Andover, MA, USA). The inhibitors in monomers were removed by a column filled with basic aluminum oxide, and the purified monomers were preserved at −20 °C in a refrigerator.

### 2.2. Synthesis of Pd NPs

The Pd NPs were synthesized according to the literature [48]. Briefly, palladium acetate (0.20 g, 0.89 mmol), n-dodecyl sulfide (1.65 g, 4.45 mmol) and toluene (50 mL, 470 mmol) were added into a three-neck round bottom flask. The reaction was stirred at 95 °C for 3 h under the protection of nitrogen. The color of the mixture changed from orange to brownish black, indicating the formation of Pd NPs. This Pd NP dispersion in toluene was directly used for later polymerization of MMA and other monomers since the toluene solution is very stable and the Pd NP is too small (~3.4 nm) to be separated by centrifugation.

### 2.3. Synthesis of Ultrahigh Molecular Weight PMMA Using Organic Halides as Initiators in the Presence of Pd NPs

The general procedure for the synthesis of PMMA using halides as initiators in the presence of Pd NPs is as follows. A sealed reaction tube charged with a stirring bar was degassed by applying high vacuum and backfilling with nitrogen. MMA purged with nitrogen was added into the reaction tube using a syringe under the protection of nitrogen, followed by the addition of organic halides (e.g., EBiB) and synthesized Pd NP solution using microsyringes. Then, the reaction tube was transferred into an oil bath set at a designated temperature. The reaction mixture was withdrawn with a syringe equipped with a long needle at different time intervals, and the obtained samples were stored in a fridge for later determination of monomer conversion, gel permeation chromatography (GPC) analysis, and ^1^H NMR measurement.

### 2.4. Synthesis of PMMA by Free Radical Polymerization

A typical free radical polymerization of MMA is as follows. Seven milligrams of benzoyl peroxide (BPO) was added into a sealed reaction tube charged with a stirring bar. The polymerization system was degassed by applying high vacuum and backfilling with nitrogen, and then the MMA (5.0 mL, 0.047 mol) purged with nitrogen was added into the reaction tube. The reaction tube was transferred into an oil bath set at 70 °C and 50 μL of the reaction solution was withdrawn by a syringe equipped with a long needle at the reaction time of 40 min for ESR measurement.

### 2.5. Synthesis of PMMA by Atom Transfer Radical Polymerization

Typically, CuBr (10.0 mg, 0.070 mmol) was added into a sealed reaction tube charged with a stirring bar. The reaction tube was degassed by applying high vacuum and backfilling with nitrogen. MMA (5.0 mL, 0.047 mol) purged with nitrogen was added into the reaction tube, followed by the addition of EBiB (4.0 μL, 0.027 mmol) and PMDETA (13.8 μL, 0.070 mmol). The reaction tube was transferred into an oil bath set at 70 °C and 50 μL of the reaction solution was withdrawn with a syringe equipped with a long needle at the reaction time of 40 min for ESR measurement.

### 2.6. Characterization

The morphology of synthesized Pd NP was determined by a Tecnai G2 F30 transmission electron microscope (TEM, acceleration voltage 300 kV, FEI, Hillsboro, OR, USA). The TEM is equipped with an energy dispersive X-ray spectrometer (EDX) accessory and has a point resolution of 0.20 nm and a line resolution of 0.10 nm. The prepared Pd NP toluene solution was washed with an equal volume of methanol and then centrifuged at 10,000 rpm for 20 min. The produced Pd NP precipitation was redispersed in tetrahydrofuran (THF) under ultrasonication. A drop of THF solution was dripped on TEM copper grid and dried for observation.

To observe the morphology of Pd NPs collected during the polymerization of MMA, the polymerization of MMA initiated by EBiB was stopped with a low monomer conversion (~5%). One milliliter of the sample was diluted in an equal volume of THF and then centrifuged at 10,000 rpm for 10 min. The bottom droplet was dripped on TEM copper grid and dried for observation.

Gel permeation chromatography (GPC) using tetrahydrofuran (THF) as an eluent at a flow rate of 1.0 mL/min at 35 °C was employed to determine the number-average molecular weight (*M*_n_), weight-average molecular weight (*M*_w_), and polydispersity index (PDI) of synthesized PMMA and other polymers. The GPC measurement was performed on a Malvern Viscotek 270 max triple detection system equipped with Viscotek T6000M GPC column (8.0 × 300 mm, molecular weight range: 1.0 × 10^3^–2.0 × 10^7^ Da), Viscotek VE1122 solvent transfer unit, Viscotek 270 laser light scattering-differential viscometer (Malvern Panalytical, Malvern, England) double detector and Viscotek VE 3580 refractive index detector (Malvern Panalytical, Malvern, England).

The ^1^H NMR of polymer was investigated by a Bruker Avance III 500 MHz spectrometer (Bruker, Fällanden, Switzerland). A polymer sample with a relatively low molecular weight was obtained by precipitation using THF as a solvent and n-hexane as a precipitant. After the polymer was dried under vacuum, it was dissolved in chloroform-d (CDCl_3_) with tetramethylsilane (δ = 0 ppm) as an internal standard for NMR measurement. The monomer conversion was determined by the gravimetric method.

The exact molecular weight of PMMA sample was determined by an Autoflex speed matrix-assisted laser desorption ionization time of flight (MALDI-TOF) mass spectrometer (Bruker, Billerica, MA, USA). The instrument was operated in a positive ion mode with an acceleration voltage at 20 kV. A peptide standard was used to verify the instrument. Trans indole-3-acrylic acid was used as a sample matrix. The PMMA, matrix and sodium trifluoroacetate were dissolved in THF at concentrations of 2.0, 20.0, and 1.0 mg/mL, respectively. Then, the three solutions were mixed together at a volume ratio of 10:10:1. One microliter of the mixture was dropped on a sample plate and air-dried at room temperature.

The valence of the Pd NPs collected during the polymerization was detected by X-ray photoelectron spectroscopy (XPS) (Shimadzu, Kyoto, Japan). Five milligrams of collected Pd NPs were spread on the surface of a silicon wafer (1 × 1 cm^2^) and the XPS characterization was proceeded using a Kratos AXIS Ultra DLD spectrometer (Shimadzu, Kyoto, Japan) with a monochromatic Al target X-ray source. The pressure of the sample analysis chamber and sample processing chamber was maintained below 5 × 10^−10^ and 5 × 10^−9^ Torr, respectively.

The radical intermediate in the polymerization was detected by a Bruker A300 electron spin resonance (ESR) spectrometer (Bruker, Karlsruhe, Germany). The instrument was worked in X-band with a microwave power of 20.39 mW and frequency at 9.8374 GHz. PBN was used as a free radical trapping agent and added into the reaction solution (PBN final concentration: 0.075 mol/L) to capture the polymer propagating chain radicals during the polymerization of MMA at reaction time of 40 min. Fifty microliters of the polymer solution was sealed into a 1.3 mm capillary tube for ESR detection. According to the literature [49,50], the ESR spectrometer was calibrated using DPPH as a standard and the radical concentration had been determined using DPPH as an external standard.

## 3. Result and Discussion

### 3.1. Characterization of Pd NPs

**Figure 1:**
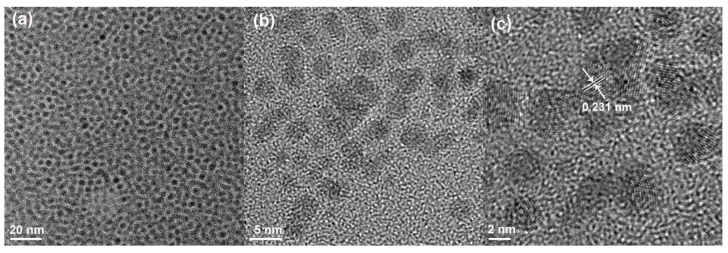
Low-resolution image of palladium nanoparticles (Pd NPs) (**a**), and lattice fringes of Pd NPs at high resolutions (**b**,**c**).

The TEM images of synthesized Pd NPs are shown in Figure 1. Figure 1a is the low-resolution image of Pd NPs, while Figure 1b,c present the lattice fringes of Pd NPs at high resolutions. Clearly, the Pd NPs were narrowly dispersed and the average diameter was determined to be 3.4 ± 0.68 nm. According to the high-resolution TEM image (Figure 1c), the interplanar spacing of synthesized Pd NPs was measured to be 0.231 nm, which corresponds to the Pd crystal (111) plane [51].

### 3.2. Synthesis of Ultrahigh Molecular Weight PMMA Using EBiB as an Initiator in the Presence of Pd NPs

The Pd NPs combined with a series of organic halides including ethyl α-bromophenylacetate (BPA), ethyl 2-bromopropionate (EBP), 2-bromoisobutyric acid ethyl ester (EBiB), and carbon tetrachloride (CCl_4_) were screened as initiators to induce bulk polymerizations of MMA and other vinyl monomers. The results are presented in Table 1. Obviously, BPA and EBP initiated slow polymerization of MMA and produced PMMA with relatively low molecular weights (Table 1, entry 2, 3). In comparison with CCl_4_ (Table 1, entry 1), EBiB promoted faster polymerization of MMA and resulted in PMMA with ultrahigh molecular weight (1.32 × 10^6^ Da) under the same reaction conditions (Table 1, entry 4). Decreasing the concentration of EBiB and Pd NP definitely slowed down the polymerization but significantly improved the molecular weight of PMMA (Table 1, entry 5). An ultrahigh molecular weight PMMA with a number-average molecular weight (*M*_n_) as high as 4.65 × 10^6^ Da and a weight-average molecular weight (*M*_w_) as high as 8.08 × 10^6^ Da was obtained at [MMA]/[EBiB]/[Pd NP] = 1.05 × 10^5^:76:1 and a reaction temperature of 70 °C (Table 1, entry 5). Further increasing the temperature to 80 and 90 °C enhanced the polymerization rate but caused a slight decrement in PMMA molecular weights (*M*_n_ ≥ 3.00 × 10^6^ Da, entry 6, 7). It is reasonable for the polymerization to proceed faster at higher temperatures according to the Arrhenius equation, but the chain transfer and chain termination rate constants also increase with the increase in temperature, resulting in a decrease in PMMA molecular weight. These results indicate that the combination of EBiB with catalytical amount (10.1 ppm, calculated from the [MMA]/[EBiB]/[Pd NP] ratio) of Pd NPs provided a facile and novel approach for the synthesis of ultrahigh molecular weight PMMA at mild conditions. The combination was also found to polymerize MA, BA, and VAc (Table 1, entry 8, 9, and 10) at mild conditions, suggesting that EBiB/Pd NP was also versatile for other vinyl monomers.

### 3.3. Kinetics of the Polymerization of MMA Initiated by EBiB in the Presence of Pd NPs

**Figure 2:**
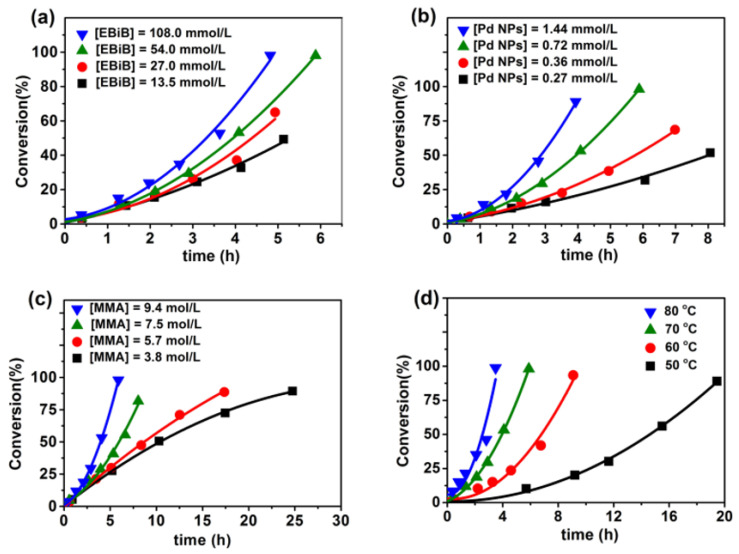
Conversion-time plots of the polymerization of MMA at different 2-bromoisobutyric acid ethyl ester (EBiB) concentrations (**a**), Pd NP concentrations (**b**), monomer concentrations (**c**), and different temperatures (**d**). Reaction conditions: (**a**) [MMA] = 9.4 mol/L, [Pd NP] = 0.72 mmol/L, T = 70 °C; (**b**) [MMA] = 9.4 mol/L, [EBiB] = 54.0 mmol/L, T = 70 °C; (**c**) [EBiB] = 54.0 mmol/L, [Pd NP] = 0.72 mmol/L, T = 70 °C; (**d**) [MMA] = 9.4 mol/L, [EBiB] = 54.0 mmol/L, [Pd NP] = 0.72 mmol/L.

To investigate the nature of the polymerization of MMA initiated by EBiB in the presence of Pd NPs, a kinetical study was systematically performed. Figure 2 shows the effects of EBiB concentration (a), Pd NP concentration (b), MMA concentration (c), and reaction temperature (d) on the rate of the polymerization of MMA. Apparently, the polymerization rate increased with the increase in EBiB concentration, Pd NP concentration and MMA concentration. According to Figure 2a–c, the initial polymerization rates determined at low monomer conversions (<10%) were used to calculate the reaction orders. The orders of polymerization with respect to EBiB, Pd NP and MMA were found to be 0.23, 0.50, and 0.58, respectively. Therefore, the reaction rate *R* could be written as *R* = −dc/dt ∝ [EBiB]^0.23^[Pd NPs]^0.50^[MMA]^0.58^. Since the reaction orders to initiator and monomer in a normal free radical polymerization are 0.5 and 1, respectively, the above kinetic results imply that the polymerization of MMA using EBiB as an initiator in the presence of a catalytical amount of Pd NPs is not a typical free radical polymerization. Figure 2d shows that the polymerization rate of MMA was significantly affected by the reaction temperature. According to Arrhenius equation, the apparent activation energy of the polymerization was determined to be 74.8 kJ/mol.

### 3.4. End Group Analysis of PMMA

**Figure 3:**
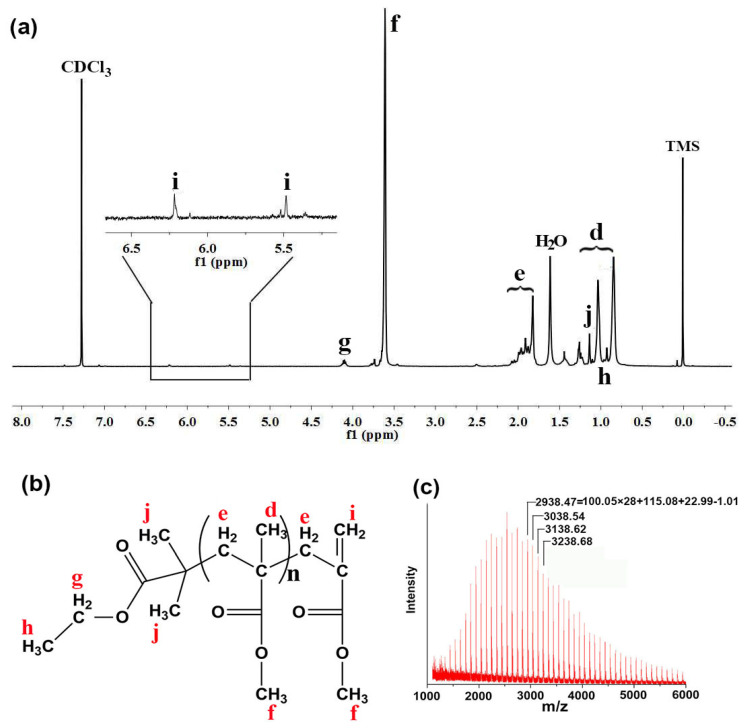
^1^H NMR spectrum (**a**), structural formula (**b**), and MALDI-TOF result (**c**) of poly(methyl methacrylate) (PMMA) prepared using EBiB as an initiator in the presence of Pd NPs.

To facilitate end group analysis of the macromolecular chain, a low molecular weight PMMA (*M*_n_ = 2620 Da, measured by GPC) was prepared at [MMA]/[EBiB]/[Pd NP] = 60:17:1 at 70 °C and then analyzed by ^1^H NMR (Figure 3a). According to the literature [52], peaks d, e, and f corresponded to the methyl (–CH_3_), methylene (–CH_2_–), and methoxyl groups (–OCH_3_), respectively, in the repeating unit of PMMA (Figure 3b). The resonance peaks of methyl group h and j of EBiB overlapped with the methyl group d of PMMA. The tiny peak g at δ = 4.08 ppm was the resonance of the methylene group of the ethyl ester group of EBiB (–OOCH_2_CH_3_). The molecular weight of PMMA was therefore calculated to be 2530 Da from the integration of peak g and peak f, which is very close to the molecular weight of 2620 Da measured by GPC, indicating that the PMMA macromolecular chain was end capped with EBiB residue. According to the literature [53,54], the peak i at δ = 5.45 and 6.19 ppm was from the proton of vinylidene group generated through disproportionation termination reactions, suggesting that the synthesized PMMA also had a terminal vinylidene structure (Figure 3b). This structure was further confirmed by matrix-assisted laser desorption ionization time of flight (MALDI-TOF) mass spectroscopy (Figure 3c). The results show that the molecular ion peaks were consistent with the Na^+^ adducts of the PMMA macromolecule chain that had a structure as illustrated in Figure 3b.

### 3.5. Mechanistic Investigation of the Polymerization Initiated by EBiB in the Presence of Pd NPs

**Figure 4:**
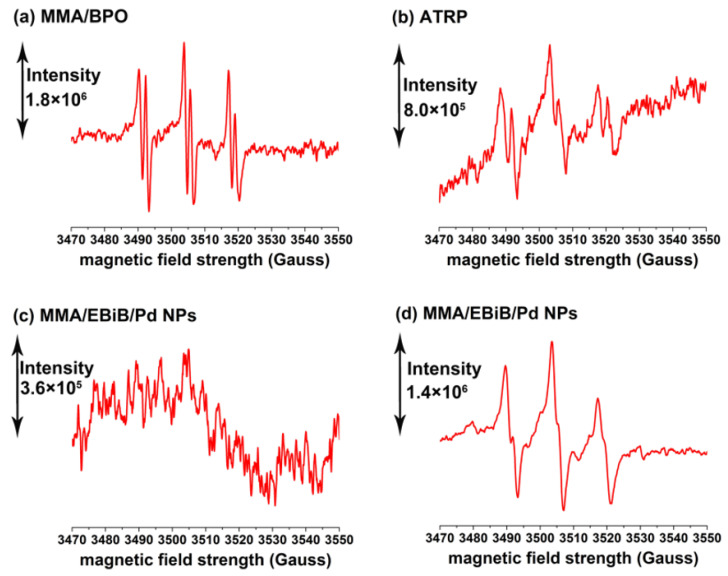
ESR spectra acquired in a free radical polymerization of MMA (**a**), atom transfer radical polymerization (ATRP) of MMA (**b**), EBiB/Pd NP-initiated polymerizations of MMA (**c**,**d**). Reaction conditions: [MMA] = 9.4 mol/L, [PBN] = 0.075 mol/L, temperature = 70 °C; all spectra acquired at reaction time = 40 min; (**a**) [MMA]/[BPO initiator] = 1.72 × 10^3^:1; (**b**) [MMA]/[initiator]/[copper catalyst] = 1.72 × 10^3^:1:2.5; (**c**) [MMA]/[EBiB]/[Pd NP] = 1.72 × 10^3^:1:0.1; (**d**) [MMA]/[EBiB]/[Pd NP] = 1.72 × 10^3^:10:0.1.

1,1-Diphenyl-2-trinitrophenylhydrazine (DPPH), a stable free radical trapping agent, was used to determine the active intermediates of the polymerization of MMA initiated by EBiB in the presence of Pd NPs. It was found that the polymerization was completely inhibited by the addition of DPPH at a concentration of 3.2 × 10^−3^ mol/L, revealing that the polymerization of MMA was basically via a radical mechanism. Electron spin resonance (ESR) using *N*-tert-butyl-alpha- phenylnitrone (PBN) as a trapping agent was further employed to identify the active species during the polymerization. Figure 4a–c show the ESR spectra of a conventional free radical polymerization of MMA conducted at [MMA]/[initiator] (molar ratio) = 1.72 × 10^3^:1, an ATRP of MMA proceeded at [MMA]/[initiator]/[copper catalyst] = 1.72 × 10^3^:1:2.5, and a EBiB/Pd NP initiated polymerization at [MMA]/[EBiB]/[Pd NP] = 1.72 × 10^3^:1:0.1, respectively. Obviously, the conventional radical polymerization presented strong typical signal of carbon-centered radicals trapped by PBN from the propagating PMMA chain [55,56], while the radical signal in ATRP was profoundly weakened because the activation/deactivation equilibrium in ATRP substantially suppressed the concentration of chain propagating radicals [16]. No appreciable radical signals were observed in the EBiB/Pd NP initiated polymerization at [MMA]/[EBiB]/[Pd NP] = 1.72 × 10^3^:1:0.1, implying that the radical concentration might be very low and close to the detection limit of the ESR instrument. The low radical concentration could significantly suppress the radical termination side reactions and thus provide a mechanistic explanation for the ultrahigh molecular weights of PMMA prepared in EBiB/Pd NP initiated polymerizations [16,57]. In order to confirm the low radical concentration in the polymerization of MMA, the EBiB concentration was increased by 10 times ([MMA]/[EBiB]/[Pd NP] = 1.72 × 10^3^:10:0.1) to improve the signal intensity. In this situation, a distinctive carbon-centered radical signal trapped by PBN was observed, as shown in Figure 4d, suggesting that the EBiB/Pd NP initiated polymerization of MMA indeed followed a radical mechanism.

**Figure 5:**
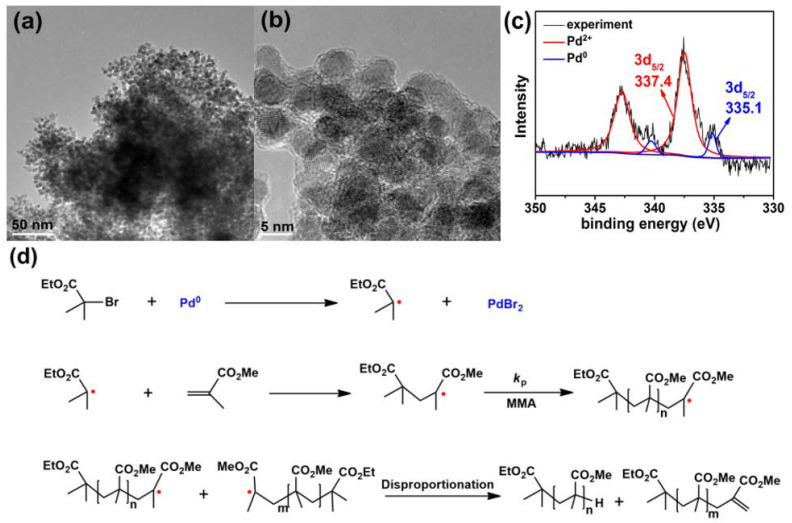
TEM images (**a**,**b**), XPS spectrum (**c**) of Pd NPs collected during the polymerization of MMA and the corresponding postulated mechanism of the polymerization of MMA (**d**).

To further reveal the origination of the radicals produced in the EBiB/Pd NP-initiated polymerization, the morphologies of Pd NPs collected during the polymerization of MMA were observed by TEM (Figure 5). Figure 5a shows that the collected Pd NPs aggregated together with irregular morphologies due to Van der Waals interactions [58,59]. Figure 5b clearly shows that there was a thin layer of amorphous polymer-like substance that appeared on the surface of Pd NPs during the polymerization. Since certain organic halides could react with activated metals (e.g., Raney metals) by one-electron transferring from metal to carbon-halogen bond to form initiating radicals [39,40,41], this result suggests that polymerization of MMA could be initiated by the radicals produced on the surface of Pd NPs via one-electron transferring from Pd NPs to C-Br bond of EBiB, followed by a fast chain propagating process in the vicinity of Pd NPs. Therefore, the chain propagating radicals were protected, in a sense, by the bulky Pd NPs. As a result, the chain radical termination and transfer side reactions were somewhat suppressed and thus ultrahigh molecular weight PMMA could be synthesized due to the suppression of radical termination and transfer reactions. The electron transferring process from Pd NPs to C-Br bond to produce EBiB residue radicals could be further confirmed by XPS spectrum of Pd NPs collected during the polymerization, as shown in Figure 5c. It clearly shows that a small amount of Pd^0^ and a large amount of Pd^2+^ with binding energy corresponding to Pd^II^Br_2_ appeared on the surface of Pd NPs [60], which suggested that the electron transferring process involved a bromo atom (Br) transfer from EBiB to Pd NPs, resulting in the generation of EBiB residue radical to initiate the polymerization of MMA and the formation of Pd^II^Br_2_ on the surface of Pd NPs. Therefore, a postulated mechanism for the polymerization of MMA initiated by EBiB in the presence of Pd NPs was proposed in Figure 5d, and a detailed mechanistic study on the polymerization of MMA is still in progress.

## 4. Conclusions

In this paper, a facile and versatile approach for the synthesis of ultrahigh molecular weight PMMA at mild conditions has been developed by the polymerization of MMA using EBiB as an initiator in the presence of a catalytical amount (10.1 ppm) of Pd NPs. An ultrahigh molecular weight PMMA with a number-average molecular weight of 4.65 × 10^6^ Da and a weight-average molecular weight of 8.08 × 10^6^ Da has been prepared at [MMA]/[EBiB]/[Pd NP] = 1.05 × 10^5^:76:1 at 70 °C. The orders of polymerization with respect to EBiB, Pd NP and MMA were found to be 0.23, 0.50, and 0.58, respectively. It was found that the polymerization of MMA was initiated by the radicals produced from the reaction of EBiB with Pd NPs. The reaction involved a bromo atom (Br) transfer reaction from EBiB to Pd NPs, which caused the generation of EBiB residue radical to initiate the polymerization and the formation of Pd^II^Br_2_ on the surface of Pd NPs.

## Figures and Tables

**Table 1:** Bulk polymerizations of methyl methacrylate (MMA) and other vinyl monomers initiated by organic halides in the presence of Pd NPs ^1^.

Entry	Mono.	Init.	Mono./Init./Pd NP (Molar Ratio)	*T* (°C)	Time (h)	Conv. (%)	*M*_n_ (Da)	*M*_w_ (Da)	PDI
1	MMA	CCl_4_	1.31 × 10^4^:76:1	70	6.0	86.6	8.84 × 10^5^	1.59 × 10^6^	1.80
2	MMA	BPA	1.31 × 10^4^:76:1	70	3.1	32.5	2.56 × 10^5^	5.66 × 10^5^	2.21
3	MMA	EBP	1.31 × 10^4^:76:1	80	5.9	38.3	7.96 × 10^5^	1.72 × 10^6^	2.16
4	MMA	EBiB	1.31 × 10^4^:76:1	70	6.0	93.0	1.32 × 10^6^	2.61 × 10^6^	1.98
5	MMA	EBiB	1.05 × 10^5^:76:1	70	24.0	82.8	4.65 × 10^6^	8.08 × 10^6^	1.73
6	MMA	EBiB	1.05 × 10^5^:76:1	80	24.0	89.0	3.96 × 10^6^	7.25 × 10^6^	1.83
7	MMA	EBiB	1.05 × 10^5^:76:1	90	24.0	91.2	3.00 × 10^6^	6.06 × 10^6^	2.02
8	MA	EBiB	1.56 × 10^4^:76:1	70	3.0	91.6	1.32 × 10^6^	2.72 × 10^6^	2.06
9	BA	EBiB	9.90 × 10^3^:76:1	70	3.0	83.0	5.77 × 10^5^	1.15 × 10^6^	1.99
10	VAc ^2^	EBiB	9.71 × 10^2^:4.8:1	80	24.0	43.7	1.66 × 10^4^	3.86 × 10^4^	2.33

^1^ Mono. = monomer, Init. = initiator; [MMA] = 9.4 mol/L, [MA] = 11.0 mol/L, [BA] = 6.9 mol/L, [VAc] = 10.8 mol/L. ^2^ The polymerization of VAc was performed in a Ace high pressure tube.
